# Characterization of the astacin family of metalloproteases in *C. elegans*

**DOI:** 10.1186/1471-213X-10-14

**Published:** 2010-01-28

**Authors:** Ja-On Park, Jie Pan, Frank Möhrlen, Marcus-Oliver Schupp, Robert Johnsen, David L Baillie, Richard Zapf, Donald G Moerman, Harald Hutter

**Affiliations:** 1 Department of Biological Sciences, Simon Fraser University, Burnaby, BC, Canada; 2 Department of Zoology, University of Heidelberg, Heidelberg, Germany; 3 Department of Molecular Biology and Biochemistry, Simon Fraser University, Burnaby, BC, Canada; 4 Department of Zoology, University of British Columbia, Vancouver, BC, Canada

## Abstract

**Background:**

Astacins are a large family of zinc metalloproteases found in bacteria and animals. They have diverse roles ranging from digestion of food to processing of extracellular matrix components. The *C. elegans *genome contains an unusually large number of astacins, of which the majority have not been functionally characterized yet.

**Results:**

We analyzed the expression pattern of previously uncharacterized members of the astacin family to try and obtain clues to potential functions. Prominent sites of expression for many members of this family are the hypodermis, the alimentary system and several specialized cells including sensory sheath and sockets cells, which are located at openings in the body wall. We isolated mutants affecting representative members of the various subfamilies. Mutants in *nas-5*, *nas-21 *and *nas-39 *(the BMP-1/Tolloid homologue) are viable and have no apparent phenotypic defects. Mutants in *nas-6 *and *nas-6; nas-7 *double mutants are slow growing and have defects in the grinder of the pharynx, a cuticular structure important for food processing.

**Conclusions:**

Expression data and phenotypic characterization of selected family members suggest a diversity of functions for members of the astacin family in nematodes. In part this might be due to extracellular structures unique to nematodes.

## Background

Astacins are a family of zinc metalloproteases. There are several hundred astacins identified in a variety of different species ranging from bacteria to humans (see [1,2] for review). The first member of this family, a digestive enzyme, was identified in the crayfish *Astacus astacus *[3]. A second member of the family, bone morphogenetic protein 1 (BMP-1), was found in vertebrates as a bone-inducing factor [4,5], illustrating the range of physiological functions associated with these proteases. BMP-1 and its *Drosophila *homologues, *Tolloid *and *Tolloid-like *are among the best characterized members of the family (see [6] for a recent review). BMP-1/*Tolloid *is conserved in evolution and found even in cnidarians [7]. In vertebrates it is involved in processing components of the extracellular matrix, most notably fibrillar collagens, where it acts as procollagen C-protease [8]. Additional substrates are TGF-β inhibitors like chordin/SOG. Cleavage of chordin by BMP-1 in the embryo leads to activation of the TGF-β signaling pathway. This has been studied extensively in *Drosophila*, where activation of the TGF-β *decapentaplegic *(dpp) on the dorsal side is a key event in patterning the dorso-ventral axis [9]. In vertebrates BMP-1 plays an additional role in the activation of two particular members of the TGF-β family. It directly cleaves the prodomain of myostatin and GDF11, leading to activation of these growth factors [10,11].

A subgroup within the astacin family are meprins, which are confined to vertebrates and found in the small intestine, kidney and skin, where they are thought to cleave biologically active peptides, cytokines and components of the extracellular matrix [12]. The discovery of the close relationship between meprin and the crayfish astacin led to the proposal to name this group of zinc metalloproteases "the astacin family" [13]. The remaining astacins form a rather diverse group including digestive enzymes, hatching enzymes and also the majority of the astacins found in *C. elegans *[3,14]. *C. elegans *astacins have been clustered into six subgroups based on their domain organization [14], specifically on domains found in the C-terminal extensions adjacent to the catalytic site. Members of subgroup I (*nas-1 *to *nas-5*) have no additional domains and subgroup II (*nas-6 *to *nas-15*) is characterized by the presence of SXC/ShK toxin domains. Members of subgroup III (*nas-16 *to *nas-30*) typically have a single EGF domain and a single CUB domain. Subgroup IV (*nas-31 *and *nas-32*) has a single SXC/ShK toxin domain in addition to the EGF and CUB domains, whereas members of subgroup V (*nas-33 *to *nas-38*) have a TSP1 domain instead. Subgroup VI (*nas-39*) consists of the single BMP-1/Tolloid homologue in *C. elegans*.

Only a few *C. elegans *astacins have been functionally characterized so far. *hch-1*/*nas-34 *is required for digestion of the outer eggshell and migration of a neuroblast [15,16]. *nas-36 *and *nas-37 *are required for molting [17,18]. They are expressed and probably secreted from the hypodermis and are thought to digest components of the cuticle to allow it to be shed. *dpy-31/nas-35 *mutants are embryonic lethal and have characteristic cuticle synthesis defects [19]. DPY-31 is the only *C. elegans *astacin with a likely substrate identified. DPY-31 is thought to be responsible for C-terminal cleavage of the cuticular collagen SQT-3 [19], a function reminiscent of the role of BMP-1 in cleaving fibrillar collagens in vertebrates [8]. DPY-31 from two parasitic nematodes, *H. contortus *and *B. malayi*, has been shown recently to have an evolutionary conserved function and a similar range of protease activity [20].

To begin a characterization of the remaining members of this family we first determined the expression pattern of previously uncharacterized genes. We then tried to isolate mutations in selected members of the different subfamilies and were able to obtain mutations in *nas-5,6,7,21 *and *39*, representing all but one of the previously uncharacterized subgroups. Mutant animals are viable in all cases indicating that none of these genes is essential for survival. *nas-6 *and *nas-7 *mutants show an incompletely penetrant slow growth and partial larval arrest phenotype. A more detailed examination of these animals revealed structural defects in the pharynx, suggesting a role for these genes in pharyngeal development. We were not able to detect any obvious defects in mutants in *nas-39 *mutants, the only *C. elegans *BMP-1/Tolloid homologue. The lack of phenotypes related to collagen processing or TGF-β signaling, characteristic phenotypes of its homologues in *Drosophila *and vertebrates, suggests that this gene, while structurally conserved, has functionally evolved independently in nematodes.

## Results

### Nematode astacin phylogeny

**Figure 1:**
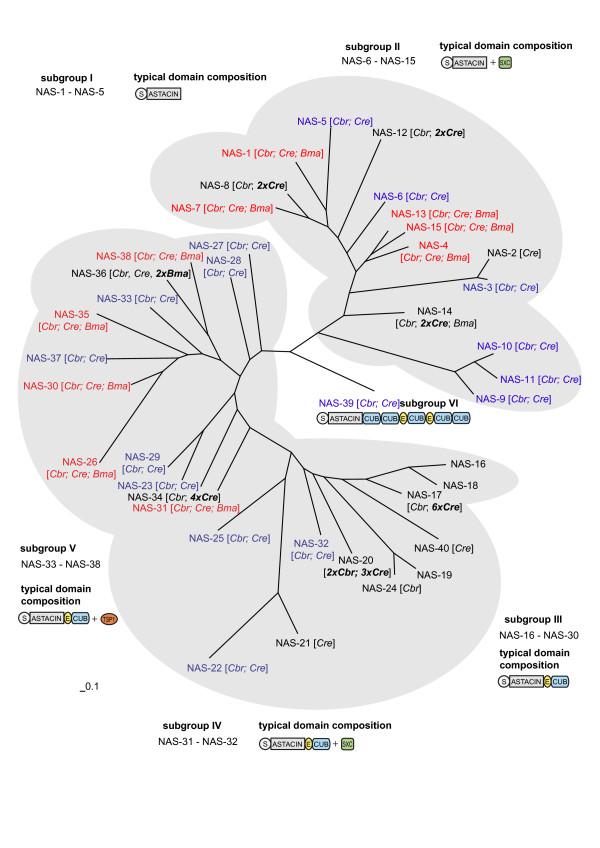
**Phylogenetic relationships of astacins within Nematodes**. The tree was deduced by Bayesian analysis based on the alignment of the amino-acid sequences of the catalytic chain, covering the region from Ala-1 to Leu-200 in the prototype, crayfish astacin. The main clusters are shaded: Cluster 1: subgroups I and II; cluster 2: subgroups III-V, The typical domain composition of each cluster is depicted. S: Pre-Pro-Sequences; Astacin: protease domain; EGF: epidermal growth factor like modules; CUB: CUB domain; TSP1: Thrombospondin type 1 domains; SXC: ShK toxin/SXC domain. The scale bar represents a distance of 0.1 accepted point mutations per site (PAM). The number of orthologs in *C. briggsae *(Cbr), *C. remanei *(Cre) and *B. malayi *(Bma) is indicated in brackets. Genes with exactly one ortholog in each of these species are in red, genes missing specifically in *B. malayi *are indicated in blue, duplicated genes in other species are in bold print.

The *C. elegans *genome contains 40 astacin genes. In a phylogenetic tree based on alignments of the protease domain subgroups I and II cluster together and also subgroups III-V (Figure 1). A comparison with sequences of other nematodes like *C. remanei*, *C. briggsae *and *B. malayi *shows that the astacin family has undergone significant evolution within the nematodes. The phylogenetic analysis points to a complex evolutionary history within the genus *Caenorhabditis *with multiple gene losses and duplications (Figure 1). The genome of *B. malayi*, a human parasite not closely related to *C. elegans*, contains only 13 astacins. These represent five of the six subgroups. Not found in *B. malayi *are a large number of members of subgroup III as well as *nas-39*/BMP1/Tolloid. Recently the genome sequences of several other nematodes have become available [21-23]. A preliminary analysis reveals the presence of about 53 astacins in *Pristionchus pacificus*, about 30 in *Meloidogyne hapla *and about 37 in *Meloidogyne incognita*. This would support the idea of a more general expansion of this protein family within nematodes. The recently sequenced genomes of the flatworms *Schistsoma mansoni *[24] and *Schistosoma japonicum *[25] (phylum: Platyhelminthes) each contain only two astacins, orthologs of *nas-4 *and *nas-39 *(Additional file 1, Table S1).

### Astacins are expressed predominantly in tissues exposed to the outside environment

**Figure 2:**
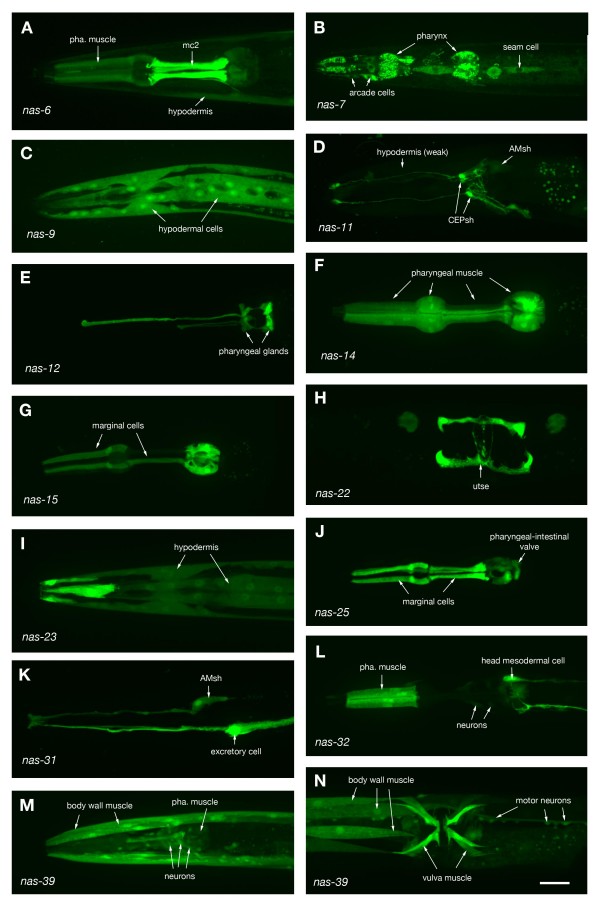
**Astacin expression**. Representative expression patterns of astacins are shown. Panels H and N show ventral aspects of the midbody regions, all other panels show side views of head regions. Anterior is to the left. Scale bar 20 μm.

We used two independent approaches to identify sites of expression for previously uncharacterized members of the astacin family: 'green fluorescent protein' (GFP) reporter constructs (Figure 2; Tables 1, 2) and serial analysis of gene expression (SAGE) of different developmental stages and embryonic tissues (Tables 3, 4). GFP reporter constructs were generated by fusing putative promoter regions with a cDNA encoding GFP. Transgenic animals were assayed for GFP expression. GFP reporters for eight of the genes gave no detectable expression (see Table 2). Some of the remaining astacins showed expression in multiple tissues, but the majority of the genes were expressed in only a few cells or cell types (Table 1). Prominent sites of expression at the tissue and organ level are the digestive system (pharynx and intestine) and the hypodermis, which express a large number of astacins (Table 2). Notably underrepresented are 'internal tissues' like body wall muscle, the nervous system and reproductive organs (gonad, uterus). These tissues only express a few astacins. Expression within the pharynx is essentially confined to two cell types, muscle cells and marginal cells. Marginal cells lie between pharyngeal muscle cells and form an integral part of the pharyngeal myoepithelium. They are thought to provide continuity and strength to the epithelium. Several astacins are expressed in various interfacial cells, many of which are responsible for generating openings in the body wall. Examples are the rectal and vulval epithelium, sensory sheath and socket cells, the excretory duct cell and the uterine-seam attachment (Table 2). Five astacins are expressed in gland cells of the alimentary tract, *nas-5 *and *nas-12 *in pharyngeal glands and *nas-2*, *nas-19 *and *nas-25 *in rectal glands, suggesting a putative role as digestive enzymes.

**Table 1:** Astacin expression according to GFP reporter constructs

gene	sub group	pharynx	intestine	hypodermis	muscle	neurons	other
*nas-1*	I	mu, mc					arcade cells
*nas-2*	I		all cells				rectal glands, 2 cells in the head
*nas-3*	I	mu3-5	all cells	hyp1, seam (weak)		PDE	ILso?
*nas-4*	I	mc (weak)					
*nas-5*	I	mc, glands					rectal glands, utse
*nas-6*	II	mu, mc2	all cells	major hyp	body wall muscle		
*nas-7*	II	mu, mc	all cells	seam (strong), other hyp (weak)			arcade cells, spermatheca, vulva, rectal epithelial cells,
*nas-9*	II			major hyp			uterus, spermatheca
*nas-11*	II		anterior most cells	major hyp			CEPsh, AMsh, PHsh
*nas-12*	II	glands					
*nas-13*	II					IL2L/R 1 pair of amphid neurons	
*nas-14*	II	mu, mc					
*nas-15*	II	mc mu3-6					
*nas-16*	III		anterior most cells				
*nas-19*	III	mu, mc	all cells				rectal glands, spermatheca
*nas-21*	III		all cells	major hyp (weak)			utse, gonad
*nas-22*	III						utse
*nas-23*	III	mu (weak)	weak, all cells	major hyp			rectum
*nas-25*	III	mc					rectal gland cells, pha-int valve, arcade cells
*nas-26/toh-1*	III		weak, all cells	major hyp			uterus, vulva epithelium, AMsh, arcade cells, PHsh, rectal epithelium
*nas-27*	III		all cells	major hyp			vulva epithelium, rectal epithelium
*nas-28*	III	mu	all cells				coelomocytes
*nas-30*	III		all cells	rectal epithelial cells			
*nas-31*	IV						exc. cell, AMsh, PHsh,
*nas-32*	IV	mu			anal depressor muscle, intestinal muscle, vulva muscle	unidentified head neurons	head mesodermal cell
*nas-33*	V		all cells	tail hyp			
*dpy-31/nas-35*	V		weak, all cells	major hyp			vulva epithelium, rectal epithelium, AMsh, IL/OLso, exc. duct cell
*nas-37*	V		weak, all cells	major hyp incl. seam (weak), rectal epi. cells			vulval epithelium, rectal epithelium
*nas-39*	VI	mu	all cells		vulva muscle, bwm,	many/most neurons	

**Table 2:** Astacin expression summarized by tissue

tissue	No of genes	genes
no expression	8	*nas-8, 10, 17, 18, 20, 24, 29, 38*
		
**Major tissues**		

intestine	16	*nas-2, 3, 6, 7, 11, 16, 19, 21, 23, 26, 27, 28, 30, 33, 35, 37, 39*
hypodermis (major hyp)	10	*nas-6, 7, 9, 11, 21, 23, 26, 27, 35, 37*
muscle (pharynx)	10	*nas-1, 3, 6, 7, 14, 15, 19, 23, 28, 32, 39*
muscle (other)	3	*nas-6, 32, 39*
neurons	3	*nas-3, 13, 32,39*
reproductive system (gonad, spermatheca, uterus)	5	nas-7, 9, 19, 21, 26
glands (pharyngeal, rectal)	5	*nas-3, 5, 12, 19, 25*
		
**Interfacial epithelial cells**		

rectal epithelium	6	*nas-7, 23, 26, 27, 35, 37*
vulva epithelium	5	*nas-7, 26, 27, 35, 37*
pharyngeal marginal cells	9	*nas-1, 4, 5, 6, 7, 14, 15, 19, 25*
		
**other interfacial cells**		

arcade cells	4	*nas-1, 3, 7, 26,*
sensory sheath and socket cells	5	*nas-3, 11, 26, 31, 35,*
uterine-seam attachment	3	*nas-5, 21, 22*
excretory duct cell	1	*nas-35*

**Table 3:** Astacin expression in stage-specific SAGE libraries

gene	oocyte	embryo	L1	L2	L3	L4	adult
*nas-4*	-	-	-	-	1.05	1.05	-
*nas-7*	0.26	1.54	0.65	1.33	-	-	-
*nas-8*	-	-	1.31	-	-	-	-
*nas-9*	0.31	-	-	-	2.53	0.42	-
*nas-11*	0.07	0.11	-	0.38	4.21	0.9	0.47
*nas-14*	0.17	0.26	-	-	-	1.05	-
*nas-28*	0.52	0.77	-	-	-	1.05	-
*nas-29*	-	0.38	-	1.33	1.58	0.53	-
*nas-31*	-	-	0.65	0.67	-	-	-
*hch-1/nas-34*	11.01	5.12	-	1.78	-	2.11	-
*nas-37*	0.1	-	-	0.8	0.63	1.26	-
*nas-38*	-	-	-	1.33	-	-	-

enrichment with respect to a mixed stage library; only genes represented in mixed stage library (contains all genes with tags in any of these libraries)

**Table 4:** Astacin expression in tissue-specific SAGE libraries

gene	pharynx	intestine	hypodermis	muscle	neurons
*nas-6a*	0.36	1.99	0.45	-	0.74
*nas-7*	0.18	-	0.23	-	1.11
*nas-11a*	-	-	0.9	-	1.48
*nas-12*	0.73	-	-	0.59	-
*nas-14*	2.18	-	1.81	-	-
*nas-28*	-	-	0.9	1.19	-
*hch-1/nas-34*	0.51	0.4	3.98	1.13	1.41

enrichment with respect to an embryonic library; only genes represented in embryonic library (contains all genes with tags in any of these libraries)

Previously characterized astacins of subgroup V (*hch-1/nas-34, dpy-31/nas-35, nas-36,37*,) are all expressed in the hypodermis [16-19] and probably process cuticle components like cuticular collagens. We found eight more astacins expressed in hypodermis (Table 2). Four of those belong to subgroup II and four belong to subgroup IV, suggesting that certain members of these subgroups might also be involved in processing cuticle components.

16 astacins across almost all subgroups are expressed in the intestine, suggesting a possible role as food digestive enzymes. With the exception of *nas-16*, all of these genes are expressed outside the alimentary tract as well. Among these are the characterized astacins *dpy-31/nas-35 *and *nas-37*, for which no role in food digestion has yet been proposed [17-19]. Members of subgroup I have no domains in addition to the metalloprotease domain. They might have broad substrate specificity, a characteristic feature of food digestive enzymes. While two members of this subgroup are expressed in gland cells of the alimentary system (*nas-2 *and *nas-5*), or the gut (*nas-2 *and *nas-3*), there is also specific expression of some members in specialized cells like anterior hypodermal cells, arcade cells or even certain neurons, arguing against a simple food digestive function.

In an independent approach expression data for astacins was extracted from a number of SAGE experiments where different larval stages and individual embryonic tissues were sampled for general gene expression (Tables 3, 4). Overall only a minority of the astacins is represented in these libraries (14 out of 40). The sequencing depth of these SAGE libraries is such that genes expressed at low levels or in only a few cells are not necessarily represented in the libraries. Nevertheless we did find representation for three genes that did not give noticeable expression with GFP reporters. *nas-8 *was found in the L1 stage library, *nas-38 *in the L2 library and *nas-29 *in the embryo and several larval stages. In all cases absolute expression levels were very low with one to four tags per library.

A comparison of expression levels across developmental stages revealed stage-specific changes for three astacins. *hch-1/nas-34 *is strongly expressed in ooctyes and embryos, as expected from its function as hatching enzyme [16]. *nas-9 *and *nas-11 *are both predominantly expressed in the third larval stage. Both genes are expressed in the hypodermis and could play a role in stage-specific processing of cuticle components.

Very few astacins are represented in SAGE libraries from various embryonic tissues. *hch-1/nas-34 *is enriched in embryonic hypodermal cells, again as expected from its proposed function. The other astacins found in these libraries show no strong tissue enrichment and are generally found in those tissues that show GFP expression in the corresponding reporter strain - with the exception of a low level expression of *nas-6*, *7 *and *nas-11 *in neurons, which was not seen with the GFP reporter expression constructs.

### Functional analysis of astacin genes

#### nas-5,6 and 7

**Figure 3:**
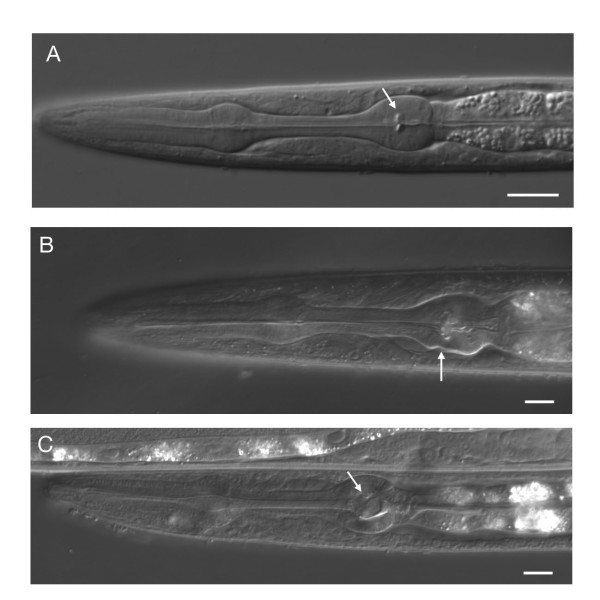
**Morphological defects in *nas-6 *mutants**. A) Head region of a wild type animal. The arrow points to the grinder in the second bulb of the pharynx. B) Morphological defects in the second bulb of the pharynx in *nas-6 *mutants (arrow). C) Morphological abnormalities of the grinder in *nas-6 *mutants (arrow). Scale bar 10 μm.

In order to study possible functions of astacins more directly we attempted to isolate deletion alleles for several members of the family across the various subgroups. We were able to isolate deletions in *nas-5,6,7,21 *and *nas-39*, members of all but one of the previously uncharacterized subgroups. All mutant strains were viable and only one had obvious defects. *nas-6(hd108) *mutant animals displayed a slow growth phenotype, with a significant fraction of the animals not reaching the L4 stage at normal speed (Table 5). About 10% of the animals arrested development at various larval stages and never reached the adult stage. Upon closer examination we detected defects in the pharynx of those more slowly growing or arrested animals. Minor morphological abnormalities were apparent in the terminal bulb of the pharynx (Figure 3B). More significantly, the grinder, a cuticular tooth-like specialization in the lumen of the terminal bulb, looked highly abnormal in slow-growing animals (Figure 3C). The role of the grinder is to grind up food (bacteria) before it is passed to the intestine. The abnormalities in the grinder probably do not allow an efficient processing of the food, which would explain the slow growth or even arrest. Consistent with this idea we find reduced pharyngeal pumping rates in slow growing animals (Table 6). The degree of visible abnormality in the grinder correlates with the slow growth or arrest phenotype (stronger grinder defects correlating with stronger growth defects). Grinder defects are apparent in embryos suggesting that the defect is developmental in origin.

**Table 5:** slow larval growth in astacin mutants

Genotype	animals hatching within 24 hours		animals reaching L4 stage within 48 hours after hatching	
wild type	99%	(n = 494)	100%	(n = 94)
*nas-5(hd96)*	97%	(n = 440)	100%	(n = 111)
*nas-6(hd108)*	98%	(n = 214)	33%*	(n = 205)
*nas-7(hd116)*	100%	(n = 208)	98%	(n = 129)
*nas-5; nas-6*	98%	(n = 257)	31%*	(n = 232)
*nas-5; nas-7*	100%	(n = 115)	100%	(n = 115)
*nas-6; nas-7*	99%	(n = 260)	17%*	(n = 135)
*nas-5; nas-6; nas-7*	96%	(n = 164)	14%*	(n = 124)
*nas-21 (hd119)*	98%	(n = 508)	100%	(n = 628)
*nas-39(hd104)*	96%	(n = 307)	99%	(n = 650)
*nas-39(gk343)*	100%	(n = 233)	100%	(n = 634)

* significant with p < 0.01 (χ^2 ^test)

**Table 6:** Pharyngeal pumping rate (pumps/minute) in slow growing animals

Genotype	average rate	maximum rate	% animals not pumping
wild type	160 ± 13	188	0%
*nas-6*	39 ± 33	110	17%
*nas-5; nas-6*	47 ± 37	124	17%
*nas-6; nas-7*	37 ± 31	115	17%
*nas-5; nas-6; nas-7*	40 ± 38	130	27%

n = 30; animals not reaching L4 stage after 48 hours were scored

Since the slow growth phenotype in *nas-6 *mutants is incompletely penetrant and since *nas-5 *and *nas-7 *are expressed in the pharynx as well, we investigated whether double or triple mutant combinations enhance the phenotype. We found that *nas-6(hd108); nas-7(hd116) *double mutants had a significantly increased number of slow growing animals, suggesting that *nas-7(hd116) *despite having no phenotype on its own, has some role in grinder development as well. The phenotype of individual slow-growing animals does not change in *nas-6(hd108); nas-7(hd116) *double mutants judging from their morphology and their pharyngeal pumping rate. *nas-5(hd96) *does not seem to be involved in this process, since *nas-5(hd96); nas-6(hd108) *double mutants are not different from *nas-6(hd108) *single mutants and since *nas-5(hd96); nas-6(hd108); nas-7(hd116) *triple mutant animals are not different from *nas-6(hd108); nas-7(hd116) *double mutants (Tables 5, 6).

#### nas-21 and nas-39

*nas-21 *is a member of subgroup III and is expressed in the major hypodermis and the uterine-seam attachment cell (Table 1). Because of this expression pattern potential phenotypes to consider would be defects in the cuticle or in the attachment of the uterus to the vulva. None of these defects or any other obvious morphological abnormalities were found in *nas-21(hd119) *mutant animals, leaving the cellular function of this gene unclear at this point. It is worth noting that *nas-22 *is structurally very similar and has an overlapping expression in both hypodermis and uterine-seam attachment. It is conceivable that there is functional redundancy between those two genes.

*nas-39 *is the only homolog of BMP-1/Tolloid in *C. elegans*. It shares the unique domain composition of five CUB domains and 2 EGF modules following the metalloprotease domain. *Drosophila *and vertebrates homologs play prominent roles in embryonic development most notably in TGF-β signaling and processing of ECM components. Mutants for *Bmp1 *in mouse or *Tolloid *in *Drosophila *are lethal. In contrast, *nas-39(hd104) *mutant animals are viable and show no obvious developmental defects. The zinc coordination site in the catalytic centre of the metalloprotease domain is deleted in *nas-39(hd104)*, so that this mutation should result in a non-functional protease and hence represent a null allele. A second allele *nas-39(gk343) *eliminates the first exon and is also expected to be a null allele. *nas-39(gk343) *mutant animals also display no obvious defects. TGF-β signaling in *C. elegans *is involved in several developmental processes and mutants in the various TGF-β genes have characteristic defects such as constitutive dauer formation [26], a reduced body length [27] or uncoordinated movement due to axon navigation defects [28]. Neither of the *nas-39 *alleles shows any of these defects, suggesting that TGF-β signaling is not affected in *nas-39 *mutants. The overall structure of the nervous system was examined in more detail with a pan-neuronal marker in *nas-39(hd104) *mutant animals. We were not able to detect significant defects in the arrangement of neuronal cell bodies or obvious axon guidance defects, suggesting that these animals do not have major neuronal defects. The function of this strikingly conserved gene in *C. elegans *currently is unclear.

## Discussion

### Nematode astacin phylogeny

The astacin family of metalloproteases with 40 members present in *C. elegans *has expanded in nematodes more than in any other phylogenetic group [14]. Orthologs for many of these genes are present in other members of the genus *Caenorhabditis*, like *C. remanei *or *C. briggsae*, suggesting that the major expansion of this family did not occur very recently, *i.e. *not within the *C. elegans *lineage itself. This can be contrasted to the current annotation of the parasitic nematode *B. malayi *which contains only 13 astacin genes. This suggests that either there was a dramatic expansion of astacins within the lineage leading to the genus *Caenorhabditis *or that *B. malayi *has lost members of this family. Gene loss within nematodes seems to be a frequent phenomenon [29] and the overall number of genes in the *B. malayi *genome is estimated to be significantly smaller than in *C. elegans *or *C. briggsae *[30,31], which supports the second hypothesis. A large number of astacins in other nematodes like *Pristionchus pacificus*, *Meloidogyne hapla *and *Meloidogyne incognita *and a small number in members of other phyla like *Schistosoma mansoni *also point to a more general expansion of the astacin family within nematodes. Details of the evolutionary history currently remain unclear because of the limited number of complete nematode genomes currently available.

### Astacin expression and function in nematodes

Only four of the astacins in *C. elegans *have so far been functionally characterized. They are required for either digesting egg-shell [15,16], shedding old cuticle during molting [17,18] or processing a cuticle component [19]. All four genes belong to subgroup V and are expressed in the hypodermis, the tissue responsible for cuticle synthesis and turnover. Site of expression and structural features defining the subgroups apparently are good predictors of potential functions for these genes.

With this idea in mind we started to characterize the expression of the remaining astacins. Expression was mainly analyzed by using reporter constructs under the control of the putative promoter regions of the gene. While this strategy greatly simplifies expression analysis and allows high-throughput studies [32], one has to keep in mind that reporter constructs do not always faithfully recapitulate the expression of the native gene. In cases where there is a discrepancy between reporter gene expression and other expression data (e.g. SAGE data) it is probably wise to consider that the reporter gene expression may be problematic. Similarly our observation that some reporter constructs did not result in any visible GFP expression most likely points to lack of essential control elements in the reporter construct rather than a genuine lack of expression of the corresponding gene. Keeping these limitations in mind, the observed gene expression patterns allow us to assess tentative sites of action and provide suggestions for potential functions.

#### Putative cuticle-components processing enzymes

The comparison of expression pattern within subgroups does not confirm a simple functional subdivision of these genes along the structurally defined subgroups. Expression in the major hypodermal cells - typical of the characterized members of subgroup V mentioned above - is found in ten astacins belonging to subgroups II, III and V. In a previous study one of the uncharacterized members of subgroup V (*nas-38*) was found to be expressed in hypodermal cells as well [32]. Two of the genes with hypodermal expression, *nas-9 *and *nas-11*, are upregulated in the L3 stage according to the SAGE data and could therefore be involved in stage-specific processing of cuticle components. The other hypodermal astacins do not show any stage-specific expression and might function in several or even all stages. *nas-9 *was found in one RNAi experiment to cause a low penetrance of embryonic lethality [33] and in a different experiment RNAi against *nas-11 *resulted in retarded growth [34]. The relevance of these results with respect to the function of these genes currently is unclear.

#### Putative digestive enzymes

The digestive tract consisting of pharynx and intestine is another major hub of astacin expression. As with hypodermal expression there is little correlation between subgroups and expression and we find members of almost all subgroups expressed here. Some of these astacins might be food digestive enzymes. Known digestive enzymes among astacins typically have no additional domains besides the protease domain [3]. *C. elegans *astacins of this type fall into category I (*nas-1,2,3,4,5*). *nas-2 *and *nas-3 *are expressed in the intestine and *nas-5 *is expressed in pharyngeal glands, promoting those three astacins as the most promising digestive enzyme candidates. Three additional astacins (*nas-12,19,25*) are also expressed in either pharyngeal or rectal glands and might also function in digestion.

#### Putative basement membrane processing enzymes

A surprisingly large number of astacins are expressed in the marginal cells of the pharynx. These cells are sandwiched between the pharyngeal muscle cells and provide continuity across the musculature of the pharynx. These cells face the lumen of the pharynx on one side and the basement membrane surrounding the pharynx on the opposite side. It is unclear whether astacins produced by these cells are secreted towards the luminal side or towards the basement membrane. Currently there is no evidence that marginal cells produce digestive enzymes, so it seems more likely that marginal cell astacins are secreted towards the basement membrane and involved in processing basement membrane components of the pharynx.

Muscle cells in *C. elegans *produce major basement membrane components including collagen and laminins, which are known substrates of astacins in other animals. Ten astacins are expressed in pharyngeal muscle cells and three are expressed in body wall muscle. These astacins potentially cleave components of the basement membrane.

#### Astacins in interfacial cells

Internal organs like the nervous system, body wall muscle cells and the reproductive system express only a small number of astacins. In contrast, a large number of astacins are expressed in a variety of interfacial cells, in particular cells associated with openings in the body wall, like rectal epithelial cells, sensory sheath and socket cells or the arcade cells of the pharynx. It is conceivable that astacins expressed in these cells have an active role in generating openings in the body wall through local breakdown of components of the cuticle and/or basement membrane.

#### nas-6 and nas-7 in pharynx development

Mutations in *nas-6 *lead to characteristic defects in pharynx development, most notably abnormalities in the grinder, a cuticular structure required for food processing. The simplest explanation for the defects is to assume a role for *nas-6 *in the processing of cuticular grinder components. *nas-6 *is expressed in pharyngeal muscle and marginal cells, which are close to the grinder, supporting this idea. Neither the molecular composition of the grinder nor its development is known in any detail. The nature of putative substrates for NAS-6 therefore is currently unclear. *nas-7 *seems to be required for this process as well, since *nas-6; nas-7 *double mutants show significantly more defects compared to single mutants. Grinder formation might be controlled redundantly by other astacins as well, since even *nas-6; nas-7 *double mutants only showed partially penetrant defects. A lack of mutants in most of the pharyngeal astacins currently prevents us from exploring this idea further.

#### Redundancy in function

Almost all astacins have been tested in several genome-wide RNAi screens. In addition to those astacins discussed above, only *nas-5*, *nas-7, nas-18 *and *nas-38 *have been identified with phenotypes in RNAi screens: *nas-5 *in a screen for axon navigation defects [35], *nas-7 *as having a reduced brood size or being embryonic lethal depending on the genotypic context [36], *nas-18 *as regulating fat content [37] and *nas-38 *as being involved in controlling life span [38]. These results point to a variety of different physiological roles for these secreted proteases. It should be noted that the axon guidance defects seen in the earlier RNAi screen with *nas-5 *could not be confirmed in the *nas-5 *mutants and that the slow growth and pharyngeal defects observed in *nas-6 *mutants here have not been reported in any of the published RNAi experiments. The overwhelming majority of astacins have not produced noticeable phenotypes in a number of genome-wide RNAi screens [33,34,39]. This might be simply due to the limited number of phenotypes scored in these screens, but it could also be a sign of functional redundancy within the family. In our study we found enhanced defects in *nas-6; nas-7 *double mutants compared to *nas-6 *single mutants, but no defects in *nas-7 *mutants alone. This kind of functional overlap with closely related family members might be a common phenomenon within the astacins in *C. elegans*. In particular, the lack of observable phenotypes in *nas-21 *mutants in particular at the uterine-seam junction might be due to functional overlap with *nas-22 *and/or *nas-5*, both of which are also expressed in the uterine-seam attachment cell. Further exploration of potential redundant function of astacins would require the isolation of mutants in the remaining family members, since RNAi experiments do not always recapitulate the phenotypes expected from the mutants. Furthermore, targeting more than one gene simultaneously in RNAi experiments leads to a significant drop in the effectiveness of RNAi, which makes it difficult to address functional redundancies with RNAi alone [40].

One evolutionarily conserved member of the astacin family in *C. elegans *is a unique member with no close relative. NAS-39 is the BMP-1/Tolloid homologue, which in *Drosophila *and vertebrates has important roles in TGF-β signaling and basement membrane collagen processing [6]. Developmental processes in *C. elegans *that are known to depend on TGF-β signaling like axon guidance [28] or regulation of dauer formation [26] and body length [27] are unaffected in *nas-39 *mutants, suggesting that *nas-39 *is not required to activate TGF-β signals. In *Drosophila *and vertebrates *nas-39 *homologues activate TGF-β signals by cleaving chordin/SOG, an inhibitor keeping the TGF-β in inactive form. Since there is no obvious chordin homologue in the *C. elegans *genome, the lack of TGF-β related phenotypes is maybe not entirely surprising. Similarly, fibrillar collagens and lysyl oxidases, which are major substrates for BMP-1 in vertebrates are also absent in *C. elegans*. The strong conservation of the unique domain composition of the BMP-1/Tolloid homologue is particularly striking and somewhat puzzling in this context. There are several possible evolutionary scenarios to explain this: firstly, some identified substrates in vertebrates including basement membrane components laminin and perlecan are present in *C. elegans *and could be NAS-39 substrates. Secondly, it is possible that the original substrate for the BMP-1/Tolloid protease is still present in *C. elegans *and hasn't been identified (neither here nor in other animals). Thirdly, NAS-39 might have acquired additional nematode-specific substrates before the original substrate had been lost from its genome. A good candidate for the evolutionary oldest substrate in this case is chordin, since it is found even in Cnidarians [41,42]).

## Conclusions

Expression data and phenotypic characterization of selected family members suggest a diversity of functions for members of the astacin family in nematodes. The large expansion of the astacin family in nematodes and the documented functions of those members where mutants are available suggest that the majority of these proteins has evolved within the nematode clade to process components of the extracellular matrix and cuticle. The size of the family and potential redundancy among closely related family members complicates the functional analysis of astacins, most of which still remain functionally uncharacterized.

## Methods

### Phylogenetic analysis

The *C. elegans *genome contains 40 Astacin genes (NAS-1-40, Möhrlen et al. 2003). NAS-40 was previously annotated as pseudogene but the current gene model (F54B8.15 in Wormbase release WS 198) predicts a complete protein coding sequence and has therefore been included in the phylogenetic analysis. The *C. elegans *genome contains a large duplication on chromosome V, which contains a duplicate of *nas-2*. This gene is called Y19D10A.6 and is identical to *nas-2 *at the DNA and protein level. Consequently it has not been included in this analysis. To identify orthologs in the completely sequenced Nematode genomes of *C. briggsae*, *C. remanei*, *Brugia malayi *and *Pristionchus pacificus *we used representative *C. elegans *and vertebrate astacins, or their conserved domains, as queries for BLAST searches of WormBase (WS198 for *C. briggsae *and *C. remanei*, WS207 for *B. malayi *and *P. pacificus*) and NCBI (Entrez Gene 10-30-2009 for the *Schistosoma *and *Melodoigyne *genomes).

For phylogenetic studies the active protease domains from all nematode astacins were aligned using CLUSTAL and imported into GeneDoc for further manipulation. The alignment is available form the authors upon request. Bayesian phylogenetic analyses were performed by MrBayes 3.0beta4 [43] with the WAG matrix [44], assuming a gamma distribution of substitution rates. Prior probabilities for all trees and amino acid replacement models were equal, the starting trees were random. Metropolis-coupled Markov chain Monte Carlo sampling was performed with one cold and three heated chains that were run for 80,000 generations. Trees were sampled every 10th generation. Posterior probabilities were estimated on 3,000 trees (burnin = 5,000). The tree presented here was visualised using TreeView.

### Generation of transgenic strains for expression analysis

Putative promoter regions of astacins were amplified by PCR following the strategy described in [32]. 5'-upstream regions extending either to the next gene or to a maximum of 3 kb were used. Primers used and regions amplified are described in Additional file 1, Table S2. Promoter::GFP fusions were generated by PCR-stitching [45]. Transgenic animals were generated as described [32].

### Analysis of GFP expression patterns

Mixed stage transgenic animals were examined for GFP expression using a Zeiss Axioplan II microscope. Stacks of confocal images with 0.2 to 0.5 μm distance between focal planes were recorded with a Quorum WaveFX spinning disc system. Image acquisition and analysis was done with the Volocity software package (Improvision). Cells were identified by location and cell morphology in comparison with reference images from Wormatlas http://www.wormatlas.org/. Maximum intensity projections of all focal planes were used to generate images for the figures.

### SAGE analysis

SAGE libraries were prepared and processed as described elsewhere [46]. SAGE tags were mapped to the latest stable release of Wormbase (WS190). Only tags that could be unambiguously mapped to a single gene were used. All tags mapping to the same gene were added up. Tags were normalized with respect to library size and enrichment was calculated as ratio of normalized tags in a particular library and tags in the reference library. Reference libraries used were a mixed stage library for the stage-specific libraries and a whole embryo library for the embryonic tissue libraries.

### Generation of mutants

Deletion alleles were isolated from a library of EMS-mutagenized animals using a poison primer approach to identify small deletions in certain region of the gene [47]. PCR primer sets were designed using AcePrimer [48]. Details about primers and deletions are given in Additional file 1, Table S3.

### Phenotypic characterization of mutants

Ten young adult hermaphrodites were placed on an NGM plate with E. coli OP50. After one hour, adult worms were removed and the eggs were incubated at 20°C. 24 hours after the eggs were laid, the numbers of hatched animals and the numbers of embryos that did not hatch was counted. 48 hours after the eggs were laid the numbers of total animals on the plate and the numbers of animals reaching L4 stage were counted. Pharyngeal pumping was scored under stereomicroscope for 1 minute in 30 worms, which did not reach L4 stage.

## Authors' contributions

J-O P, JP and MS generated GFP reporter strains and isolated and analyzed astacin mutants. FM characterized GFP reporter strains and did the phylogenetic analysis. RJ and DB produced the majority of the GFP reporter strains. RZ, MM, SJ and DM provided the SAGE data, HH analysed expression patterns and wrote the manuscript together with FM, DGM and JP. All authors read and approved the final manuscript.

## Supplementary Material

Additional file 1 **lists of orthologs, primer sequences and deletion alleles**. Table S1: Orthologs of *C. elegans *astacins. Table S2: Primers used to amplify promoter regions. Table S3: Details of deletion alleles used for functional analysis. Click here for file
