# Supplementary material for: Characterization of the astacin family of metalloproteases in C. elegans
Source: BMC Dev Biol. 2010 Jan 28;10:14. doi: 10.1186/1471-213X-10-14 (PMC2824743; doi:10.1186/1471-213X-10-14)
Supplement: Additional file 1 — lists of orthologs, primer sequences and deletion alleles. Table S1: Orthologs of C. elegans astacins. Table S2: Primers used to amplify promoter regions. Table S3: Details of deletion alleles used for functional analysis. [file 1471-213X-10-14-S1.DOC]

Table S1: Orthologs of *C. elegans* astacins

| **sub- group** | **gene in *C. elegans* (WS198)** | **orthologs in  *C. briggsae* (WS198)** | **orthologs in  *C. remaneii***  **(WS198)** | **orthologs in  *Brugia malayi*** | **orthologs in *Schistosoma mansoi* and *Schistosoma japonicum*** |
| --- | --- | --- | --- | --- | --- |
| I | *nas-1* | Cbr-nas-1 | Cre-nas-1 | Bm1_29860 |  |
|  | *nas-2* | **-** | CRE10794 | **-** |  |
|  | *nas-3* | Cbr-nas-3 | Cre-nas-3 | **-** |  |
|  | *nas-4* | Cbr-nas-4 | Cre-nas-4 | Bm1_30065 | Smp_047460  Sjp_0029490 |
|  | *nas-5* | Cbr-nas-5 | Cre-nas-5 | **-** |  |
| II | *nas-6* | Cbr-nas-6 | Cre-nas-6 | **-** |  |
|  | *nas-7* | Cbr-nas-7 | Cre-nas-7 | Bm1_23890 |  |
|  | *nas-8* | Cbr-nas-8 | **1: Cre-nas-8**  **2: CRE14419** | **-** |  |
|  | *nas-9* | Cbr-nas-9 | Cre-nas-9 | **-** |  |
|  | *nas-10* | Cbr-nas-10 | Cre-nas-10 | **-** |  |
|  | *nas-11* | Cbr-nas-11 | Cre-nas-11 | **-** |  |
|  | *nas-12* | Cbr-nas-12 | Cre-nas-12 | **-** |  |
|  | *nas-13* | Cbr-nas-13 | Cre-nas-13 | Bm1_48980 |  |
|  | *nas-14* | Cbr-nas-14 | **1: Cre-nas-14**  **2: CRE21080** | Bm1_31525 |  |
|  | *nas-15* | Cbr-nas-15 | Cre-nas-15 | Bm1_38890 |  |
| III | *nas-16* | **-** | - | **-** |  |
|  | *nas-17* | Cbr-nas-17 | **1: Cre-nas-17**  **2: CRE11193**  **3: CRE11278**  **4: CRE11189**  **5: CRE11192**  **6: CRE11191** | **-** |  |
|  | *nas-18* | **-** | **-** | **-** |  |
|  | *nas-19* | **-** | **-** | **-** |  |
|  | *nas-20* | **1: Cbr-nas-20**  **2: CBG23213** | **1: Cre-nas-20**  **2: CRE11277**  **3: CRE11190** | **-** |  |
|  | *nas-21* | **-** | Cre-nas-21 | **-** |  |
|  | *nas-22* | CBG23213 (pred.) | Cre11202 (pred.) | **-** |  |
|  | *nas-23* | Cbr-nas-23 | Cre-nas-23 | **-** |  |
|  | *nas-24* | Cbr-nas-24 | **-** | **-** |  |
|  | *nas-25* | Cbr-nas-25 | Cre-nas-25 | **-** |  |
|  | *nas-26/toh-1* | Cbr-toh-1 | Cre-toh-1 | Bm1_41080 |  |
|  | *nas-27* | Cbr-nas-27 | Cre-nas-27 | **-** |  |
|  | *nas-28* | Cbr-nas-28 | Cre-nas-28 | **-** |  |
|  | *nas-29* | Cbr-nas-29 | Cre-nas-29 | **-** |  |
|  | *nas-30* | Cbr-nas-30 | Cre-nas-30 | Bm1_49145 |  |
|  | *nas-40* | **-** | Cre13041 (pred.) | **-** |  |
| IV | *nas-31* | Cbr-nas-31 | Cre-nas-31 | Bm1_04145 |  |
|  | *nas-32* | Cbr-nas-32 | Cre-nas-32 | **-** |  |
| V | *nas-33* | Cbr-nas-33 | Cre-nas-33 | **-** |  |
|  | *hch-1/nas-34* | Cbr-hch-1 | **1: Cre-hch-1**  **2: CRE08443**  **3: CRE06152**  **4: CRE06149** | **-** |  |
|  | *dpy-31/nas-35* | Cbr-dpy-31 | Cre-dpy-31 | Bm1_41035 |  |
|  | *nas-36* | Cbr-nas-36 | Cre-nas-36 | **1: Bm1_07340**  **2: Bm1_00555** |  |
|  | *nas-37* | Cbr-nas-37 | Cre-nas-37 | **-** |  |
|  | *nas-38* | Cbr-nas-38 | Cre-nas-38 | Bm1_29450 |  |
| VI | *nas-39* | Cbr-nas-39 | Cre-nas-39 | **-** | Smp_134430 Sjp_0004770 |

Table S2: Primers used to amplify promoter regions (based on wormbase version 170)

| **gene** | **strain** | **primer** | **product size** | **promoter region** |
| --- | --- | --- | --- | --- |
| *nas-1* | BC13689 | fu1 GCAAATTTTCGGCAATTTTATT  fu3 AAAACATGGAAACCGACTAAAAA  fu2 GGAG[C/G]ATTATGGATCTGCAAG | 1236 | -1592 to -356 |
| *nas-2* | BC13426 | fu1 CTGCTGAAAATGATTGCAAAA  fu3 TTAAAATTTCCGGTCGCTTG  fu2 [C/G]ATAAAATCGTCAAATCGGCA | 2905 | -1940 to 965 |
| *nas-3* | VH1582 | fu1 CCGTAAAGCAGAGCCATAAAAC  fu3 TCTGTTTCCAAGTGTGTGCC  fu2 GTTAGAAGTTGGAGTGGGTGAA | 2851 | -2859 to -8 |
| *nas-4* | BC12429 | fu1 ATTTATGAATCCTGTGACCCATCT  fu3 TGTTTGGTAGGGATTATGGATTCT  fu2 GTAAATCATTTTTAAACTCGCCTG | 1194 | 1086 to -108 |
| *nas-5* | BC13656 | fu1 TGCCTGCCTACCACTGAAG  fu3 AACAAACATGCGACACACAAA  fu2 TGATGTC[C/G]ATTTTCCCTTAAAAA | 2814 | -2804 to 10 |
| *nas-6* | VH1581 | fu1 TTGCACGGGAAATTTTTATTG  fu3 TTGACACATCCCCAAGTTGA  fu2 ACTGCCATCACCACGTATCTC | 2907 | 2939 to 32 |
| *nas-7* | VH1260 | fu1 CCAACTTCTTCCCCTTTTTCTT  fu3 TCTTTTCCTTCTGCTCCTCTTTT  fu2 CTGAAACTATGCGAATGCGA | 2859 | 2867 to 8 |
| *nas-8* | BC13602 | fu1 ATGAGCTGAACTGTTCCCAATAA  fu3 GGAATAGTTGTCATTCGGGGT  fu2 TCTGCG[C/G]ATCTCGTGTTTC | 2715 | -2706 to 9 |
| *nas-9* | BC13411 | fu1 TCTGTGAAAATATTTCAAATGTGG  fu2 TGAACAGAATGATGAATTGCG  fu3 ATTTCAAATGTGGAATAAACCTGT | 722 | 639 to -83 |
| *nas-10* | BC13407 | fu1 TATCACTTCAAACATTCAGGGAGA  fu3 TCCATCAGAAGACACGAGCTTA  fu2 [C/G]ATGCCTCATAAATTTTAGAACAA | 2874 | 2872 to -2 |
| *nas-11* | BC13600 | fu1 CGTTCTGATTTCTTCCCAATTC  fu3 TCCCAATTCTATCTCGTATGTTCA  fu2 TGCTG[C/G]ATTAGTGTTAAATGCAA | 725 | -740 to -15 |
| *nas-12* | BC13601 | fu1 ATGTTTCAAATCACCTCCGAA  fu3 CCCGGTTAATAATACTTCAATACA  fu2 GCGATTTTATACCAACGGACA | 287 | -336 to -49 |
| *nas-13* | BC13678 | fu1 TTTGATCTCAGTGCAAATAAAAGG  fu3 AACTCACGCATTCCCGTAAG  fu2 GGCGGGCTAAAATAAACCA | 1318 | 1329 to 11 |
| *nas-14* | BC13572 | fu1 TCCTTTGGCATTCCAATCA  fu3 TGGGTTCGTGAATCGAAAA  fu2 AAAATGTGTACCCGAGTGACG | 2942 | -2947 to -5 |
| *nas-15* | BC13471 | fu1 TTTGCATTTCAAACAATCTTCG  fu3 TCAAACAATCTTCGCTTTTTATTC  fu2 CCGCACTT[C/G]ATGTCATGTCTA | 947 | -2460 to -1513 |
| *nas-16* | BC13681 | fu1 TTGAATTAACGACGAGTTCCG  fu3 GGATTTCCATTTGGTCCCTT  fu2 C[C/G]ATGATTATGCAGGAGGAAA | 2906 | -2902 to 4 |
| *nas-17* | BC13642 | fu1 TTGAATCATCTTTTGATCTTTTGA  fu3 TGTCGAATATTCTTTCTTCTTTTG  fu2 GATGAGTCTCTCATGATTATGCAC | 950 | -953 to -3 |
| *nas-18* | BC13468 | fu1 TTTCGTTTGGTGATTTGTTCTG  fu3 ACAACAATCAATGAATCAAACCAG  fu2 AGTTGCCCCTAAATGCCAG | 2191 | -2200 to -9 |
| *nas-19* | BC13470 | fu1 GAAGAACAGAGTGGCTCATGC  fu3 TTTGAAGGAAAGTCGGCATC  fu2 TTCACTGTACAACGAGCCCTT | 2754 | 2812 to 58 |
| *nas-20* | BC13606 | fu1 CGATTGTCGTGGAATCACTTT  fu3 GCATCACATGAACACTAACCG  fu2 GCTTGCATTTCAAGTGCTAAAA | 2896 | 2624 to -272 |
| *nas-21* | BC13466 | fu1 AAACAAACGTTCACAACAATCG  fu3 AACAATCGAATGCATTTTTCATAG  fu2 TGCAAATGCGACGAATAAAA | 2974 | -2962 to 12 |
| *nas-22* | BC13423 | fu1 GAATGGAACCAAACTTCACCA  fu3 TCGAAGTCGATATTCGAGAGA  fu2 ACTGTGCTTGCAATTGTGCTT | 1018 | -1022 to -4 |
| *nas-23* | VH1589 | fu1 TCCAGAATGTCTTGATGGAGC  fu3 TCCAATCGATCCATTTCTTCTT  fu2 TTACTTGGGATTGCAACGTCT | 1922 | 39 to 1960 |
| *nas-24* | BC13640 | fu1 GGCACAAGTGGGAGTTCATAA  fu3 TTCCCAGGATAGCGTTTGAC  fu2 TCGAGATGTTAAATGATTCTTCCA | 2915 | -2916 to -1 |
| *nas-25* | BC13461 | fu1 GAAGCTTGTTTGAAGTTGTGCTAA  fu3 CAAAAACGAAACGGAACAAAA  fu2 CTGCATAAGTGACAGGAATAACCA | 2878 | -2874 to 4 |
| *nas-26* | VH1633 | fu1 GAAAGTGCACCAAAACCTGAA  fu3 TAAGCCTAAGCCCCAGGCCTAA (n)  fu2 AGGATGTCATTGGTGCTTGAG (n) | 1104 | -190 to -1294 |
| *nas-27* | BC13467 | fu1 AAATTGCAAAATCTCTTTAGCCC  fu3 ACTATCGGAAGCAGAAAATTGG  fu2 TCGATAGGTGTTTGCGTACTTTT | 2870 | 2902 to 32 |
| *nas-28* | VH1253 | YFP1 ACATAAAAGTGGGGAAATCTCAAAAA  YFP2 CACGAAAGGGATGAAGAATACAAC | 4775 | 4741 to -34 |
| *nas-29* | BC13427 | fu1 TGGTTGCTTGCTCAAAGATG  fu3 AGTGGCCTGAAACCATTTCTT  fu2 ATAACTAATCGC[C/G]ATGTCTCCTTC | 2845 | 2048 to -797 |
| *nas-30* | BC13565 | fu1 TTGGAAAAGCACAATTCCTTTAAT  fu3 AGCACAATTCCTTTAATACAAAAA  fu2 TTTTTAGGGTATTTTGAGACAGGC | 827 | -990 to -163 |
| *nas-31* | BC13573 | fu1 TGGAACCTAAGATCCTAAACGAAG  fu3 TCCTAAACGAAGAGCATGAGTTC  fu2 GTTGCAATAT[C/G]ATGCTCTTTTGAC | 2946 | 2855 to -91 |
| *nas-32* | VH1634 | fu1 GGAAGAGTTTGGAGGGAAAGA  fu3 GGCCGAGCTACTCAACAAAC  fu2 GGTATGTTTTAATCCCGCCAT | 95 | 52 to 146 |
| *nas-33* | VH1584 | fu1 CATGGGTGGTATAATATTCAGGC  fu3 AGCCCATAGTGGCGATACTG  fu2 CTGGTTACC[C/G]ATGGAGGTCTT | 2408 | 2398 to -10 |
| *dpy-31/ nas-35* | BC14090 | fu1 CACCAGTTCTCATATCAAATTCTC  fu3 AAGGTTATAAGCTCCGCCTATTG  fu2 TTTTGTG[C/G]ATGCTGAAATTAAAGT | 2864 | -2857 to 7 |
| *nas-37* | BC13462 | fu1 CATAGCGCAAATATTGTAGGAGG  fu3 CAAATATTGTAGGAGGCAAGTCG  fu2 TGCAAAATAGAACATCAAGAATCG | 2899 | 2901 to 2 |
| *nas-38* | VH1706 | fu1 GGAAAGTTGGGAGAATTGGAG  fu3 GCGTGCAGCAACTGATAATG  fu2 GTTGTGTGATTGGCTTTTCGT | 2920 | -2923 to -2 |
| *nas-39* | BC13431 | fu1 TTATCATCCACCAATTCTTCCC  fu3 GATCTCCCCTTATATGTTTTGCC  fu2 TCGCAGAAAATCG[C/G]ATGTC | 1572 | -1529 to 43 |

Table S3: Details of deletion alleles used for functional analysis

| **allele** | **primer** | **deletion size** | **flanking region** | **consequence** |
| --- | --- | --- | --- | --- |
| *nas-5(hd96) I* | dx3: caaaaatttccagaaccgtctc dx4: ttcgaaaatgttcaaaatgcac di3: actttaagtgaaacatgccgct di4: gattgatcatcattgctctcca dp3: gtcaatggacgggtaagtgttt | 436 bp | cattttttgaaacttttcagtaaaagtgaa - DELETION - atatagttcaactacgggggcctgcagagc | eliminates exon 1 and part of exon 2 |
| *nas-6(hd108)* | dx1: gtttcaaggggatattgatgga  dx2: ttgatgaggttcctcggtagtt  di1: aattatcttgcaggaaggtcca  di2: ggtcccaataacctgtgtgaat  dp1: gagagctggtcagttcgtgtaa | 770bp | acattgtaaaaggatatgggtaagaatata-DELETION - aaatactctcgaaaaagaataaaatgaaca | eliminates exons 5-6 (most of the astacin domain including the catalytic site), resulting in frame shift |
| *nas-7(hd116)* | dx1a: cttttgccatggatcatcacta  dx2: agagatcacctgtaaggggtca  di1: aattccagaggaactgtttgga  di2: attgcaggagacctacaacgat  dp2: agagataacacctgaactcccg | 890bp | cagaggaactgtttggaaaacacattccgg-AAAAAAACACA (insertion)-DELETION-ataaatcgattcttcattatgatagtttgg | eliminates part of exon 2, exons 3-5 and part of exon 6 (most of the astacin domain) |
| *nas-21(hd119)* | dx1: gcaaatcgggttaagaaaagtcta  dx2: aatttttggatgctcttaaagtcg  di1: tttagggactcgtttggatactgt  di2: aaagttgaaagtggtgatttagcc  dp2: tgaatttttaggcccatattcatt | 1018bp  and  36bp | 1) atattgtcttgcatcgactataaaatactt-TCCAAACTTCACCAAATA(insertion)-DELETION- aaccaaacttcaccaaatagatttcgtatt  2) taaatataacattttctacagatttacgga-DELETION- ttgagataaattctatttttacttccaggc | eliminates exons 1-2 and part of exon 3 |
| *nas-39(hd104)* | F38ace_EL:aaaaacgacgtaaatgtctcgca  F38ace_ER:ttgatttttctgattggcccttt  F38ace_PL:ggggagccatatgatttctcatc  F38ace_IL:tctacctgctgaacttgactccg  F38ace_IR:gtttgacccaagctgtgtagacg | 993 bp | ccttctattgttttaaaaat [993 bp DELETION] ccggaaatttggcgattcaa | eliminates exons 3-6 (astacin domain including zinc binding motif and met turn) and part of exon 7 resulting in a frame shift |
| *nas-39(gk343)* | GK343_external_f: gcacctaacaggcattccat  GK343_external_b: tgcaaaatgtgggaaacaaa  GK343_internal_f: cccgagctcctcctcttact  GK343_internal_b: gtaaagttctcccaatgccg | 344 bp | atgagtgatctaaattgacat [344 bp DELETION] tgagaattaatttttaaaact | eliminates first exon with start codon |
